# A Semi-Automated Pipeline for the Segmentation of Rhesus Macaque Hippocampus: Validation across a Wide Age Range

**DOI:** 10.1371/journal.pone.0089456

**Published:** 2014-02-24

**Authors:** Michael R. Hunsaker, David G. Amaral

**Affiliations:** 1 Department of Psychiatry and Behavioral Sciences, University of California, Davis Medical Center, Davis, California, United States of America; 2 UC Davis MIND Institute; University of California, Davis Medical Center, Davis, California, United States of America; University of Minnesota, United States of America

## Abstract

This report outlines a neuroimaging pipeline that allows a robust, high-throughput, semi-automated, template-based protocol for segmenting the hippocampus in rhesus macaque (*Macaca mulatta)* monkeys ranging from 1 week to 260 weeks of age. The semiautomated component of this approach minimizes user effort while concurrently maximizing the benefit of human expertise by requiring as few as 10 landmarks to be placed on images of each hippocampus to guide registration. Any systematic errors in the normalization process are corrected using a machine-learning algorithm that has been trained by comparing manual and automated segmentations to identify systematic errors. These methods result in high spatial overlap and reliability when compared with the results of manual tracing protocols. They also dramatically reduce the time to acquire data, an important consideration in large-scale neuroradiological studies involving hundreds of MRI scans. Importantly, other than the initial generation of the unbiased template, this approach requires only modest neuroanatomical training. It has been validated for high-throughput studies of rhesus macaque hippocampal anatomy across a broad age range.

## Introduction

The rhesus macaque (*Macaca mulatta*) is an animal model often used to model human brain development. As an increasing number of neurodevelopmental and neurodegenerative disorders show hippocampus pathology as an early presenting feature [1], [2], [3], it has become increasingly important to reliably quantify the volumes and shape of the in vivo hippocampus both in humans and in nonhuman primates across ages (*cf.,* [4]). At present, there are only sparse data concerning the typical maturation of the nonhuman primate brain [5], [6]. Such data are crucial towards understanding the maturation of the human brain, particularly during early postnatal brain development.

There currently exists many ways to obtain hippocampal volumes. Manual segmentation, where a trained experimenter traces the hippocampus slice by slice, is the accepted gold standard for hippocampal measurement [7], [8], [9], [10]. The principle drawback to manual segmentation is the extensive time required of the operator, both in terms of the months of dedicated neuroanatomical training as well as the actually time spent performing the segmentations. Our experience is that it takes at least 45 minutes (and often well over an hour) to trace a single monkey or human hippocampus, resulting in around 2 hours of tracing per brain. The quality of manual segmentation is also highly dependent on consistent training among raters, and is subject to fluctuations due to inter- and intra-rater bias [7]. Mitigating any systematic bias or flow in tracing protocols across time typically involves tracing brains from earlier experiments as a part of every experiment along with 10% of the experimental sample to generate reliability estimates. Only upon obtaining consistent reliability across these test brains are hand tracings considered unbiased. Each tracer continues to practice tracing hippocampi until their reliability is consistently maintained across a number of brains. This requirement of establishing reliability increases the amount of time and effort required to obtain reliable data. For studies that have a small number of subjects, this may be an acceptable situation. But, in studies involving hundreds of participants, the time demands of manual tracing greatly diminish productivity.

Manual tracing may also lead to inaccuracy due to biased perceptual processes of even gold standard human tracers. For example, it has recently been demonstrated that there is a strong tendency for even experienced researchers to trace the same hippocampus as larger if it is on the right side of the computer screen (*i.e.,* volumes will be different for the same hippocampus when traced in neurological compared to radiological space; *cf*., [11]). These types of systematic human errors raise the prospect that human instructed semi-automated algorithms, which do not suffer from perceptual biases, may actually be better than the gold standard for carrying out morphometric analyses of regions of interest in MRI studies.

Several automated methods have been developed to perform segmentations more quickly. Commonly, normalization is performed between each subject and a predefined template, and then a hippocampus that is defined in template space is warped back into the native space of the subject using B-spline or other nonlinear warping methods (*cf.,* [12]). Other methods use shape matching and boundary definitions to perform the whole segmentation in native space [13], [14]. While these methods have been shown to create relatively accurate segmentations, they are particularly poorly suited to the segmentation of anatomy that has been affected by disease, injury, or aging (*e.g.*, optimal template effect [15], [16], [17]). Moreover, none of these methods have been validated in nonhuman primate models.

Previous semi-automated algorithms attempt to make segmentation of abnormal structures possible by requiring initial involvement from the user to guide the automated segmentation. Typically this involves the experimenter placing landmarks to guide the nonlinear warping of a hippocampal mask. However, many of the existing methods that have achieved a reasonable degree of agreement with manual segmentations require an impractical number of landmarks, upwards of 200 per hippocampus [18]. This results in challenges similar to those encountered with standard manual tracing including significant training requirements in hippocampal neuroanatomy and large expenditures of time. There have been reports about methods that employ fewer landmarks [19], [20], [21], but these typically return less reliable and somewhat less accurate results.

The goal of the current study was to develop an easy to use methodological pipeline that would be accessible to any research laboratory to partially automate the segmentation of anatomical regions of interest. The first goal was to use freely available tools that had similar dependencies and did not rely upon any commercial software packages to implement. The second goal was to develop a pipeline that would facilitate consistent data across laboratories by removing experimenter bias as much as possible without sacrificing neuroanatomic rigor.

We have adapted for use in the rhesus macaque model an incomplete label matching strategy for diffeomorphic template based hippocampus segmentation reported by Pluta et al. [22] originally used in human populations. This method requires fewer than a dozen landmarks per hippocampus and shows a high reliability and spatial overlap between semi-automated and manual segmentations, while providing dramatic time saving. To improve upon the semi-automated methods, the resulting hippocampus segmentations were subsequently corrected with a machine learning-based (SegAdapter) wrapper developed by Wang et al. [23]. The resulting corrected segmentations showed an increase in spatial overlap and appear to reach an asymptotic level of reliability that approaches the level of accuracy and reliability of high quality manual segmentation experiments. The advantage of this process is that it, requires much less experimenter time and thus facilitates larger sample sizes and more comprehensive analyses.

## Materials and Methods

### Ethics Statement

All work was conducted in accordance with the recommendations of the Weatherall Report “The use of nonhuman primates in research”. This study was carried out in strict accordance with the recommendations in the Guide for the Care and Use of Laboratory Animals of the National Institutes of Health. The University of California, Davis Institutional Animal Care and Use Committee approved all animal experimental protocols (Protocol Number 13483). All testing procedures were developed through consultation with the veterinary staff at the California National Primate Research Center (CNPRC). Every possible effort was undertaken to minimize animals’ stress and promote their well being.

### Subjects

Rhesus macaque monkeys (*Macaca mulatta*) were studied from birth through five years of age for behavioral and structural brain development. Naturalistic behavioral observations were conducted in their home environments regularly. At periodic intervals (1, 4, 8, 13, 26, 39, 52, 156, and 260 weeks of age), subjects were brought in from their naturalistic outdoor enclosures for behavioral tests, measurements of physical development, and MRI scans of the brain.

Twenty-eight rhesus macaque monkeys (14 males, 14 females) were selected from the CNPRC in the spring of 2007. Infants were raised in social troops by their biological mothers in outdoor, half-acre enclosures that house 70 to 155 animals. Subject selection was based on characteristics of the mother. Mothers were selected based on the following factors: (1) rank of matriline (high, n = 8; middle, n = 9; low, n = 10); (2) previous reproductive experience (multiparous, n = 25; primaparous, n = 3); (3) absence of previous medical problems such as diabetes, arthritis, etc. Three of the subjects were hospitalized during the course of the analysis for symptoms of dehydration caused by bacterial or parasitic gastrointestinal infection. These subjects were successfully treated and remained in the study. Treatment included administration of fluids and antibiotics. Two subjects were removed after 1 year of age due to recurrent illness. One subject was removed from the study at 4 months of age due to non-pathogenic diarrhea that was not responsive to treatment. Therefore, 24 subjects (n = 12 male and n = 12 female) received MRI scans at all ages and only data from these subjects will be reported in this manuscript.

Cohort characteristics occasionally changed after the selection of subjects. For example, rank shifted for multiple matrilines. So, the social rank was assessed monthly based on two, 30-minute observations by CNPRC behavioral specialists. All dyadic aggressive and displacement interactions, with and without food as a precipitating stimulus, were recorded and used to determine the hierarchy of the females in each troop. Rank status was determined to have changed when displacements (submission to a lower ranking rhesus macaque in the selection of food) were observed twice for the mother of the infant. Rank was consequently raised for the primate that displayed dominance in the food challenge. Social rank was raised for one primate (low to mid) and shifted downward for two others (mid to low) and (high to mid). For the latter two animals, this shift took place when their mothers were removed from their home enclosures after weaning and the infants remained with their respective matrilines.

Infants were born and reared by mothers that resided in large, 2000 m^2^ outdoor corrals. All seven of the corrals that housed study animals were chain-link and consisted of grass and gravel ground substrate and included a variety of hanging, climbing, and resting structures. The number of animals that lived in these corrals ranged from 70–155 individuals and all the kin relationships of the monkeys were known. Primates were fed twice per day, in the morning and afternoon, with chow (Lab Diet 5047, PMI Nutrition International Inc., Brentwood, MO) and supplemented with fresh fruit and vegetables.

### Animal Husbandry

For MRI scans collected at 1, 4, 8, 13, and 26 weeks of age, infants were relocated with their mothers and were housed together in a standard macaque indoor housing cage (61 cm in width by 66 cm in depth by 81 cm in height) one day prior to behavioral testing. On days when testing was to occur, mothers were lightly sedated with ketamine hydrochloride (7 to 8 mg/kg i.m.) and infants were removed from the cage for testing. Beginning at 39 weeks of age, each rhesus macaque subject was removed from its respective home enclosure without the mother the day prior to behavioral testing and was temporarily housed indoors as described above.

### Structural MRI Acquisition

After behavioral testing at the CNPRC, animals were transported to the Imaging Research Center (IRC) for the MRI scan. Each subject was fasted a minimum of two hours prior to sedation for scanning. Subjects were transported from the CNPRC to the IRC by van either in incubators (30.5 cm in width by 30.5 cm in depth and 30.5 cm in height; at 1, 4, 8, and 13 weeks of age) or in a transport box (31.0 cm in width by 51.0 cm in depth by 40.0 cm in height; at 26, 39, 52, 156, 260 weeks of age). Animals were anesthetized and monitored by a veterinarian at 1 and 4 weeks of age, then by an animal health technician from 8–260 weeks of age. Each macaque was sedated with ketamine hydrochloride (1 mg/kg i.m.) during catheter placement and intubation. During the scanning procedures, each rhesus macaque was anesthetized with propofol (2 ml/kg/hr i.v.). The anesthesia rates were managed remotely from the control room of the scanner suite using a Harvard Apparatus 4500 infusion pump (Harvard Apparatus; Holliston, MA). Intravenous saline was administered throughout the scanning procedure to reduce the possibility of dehydration. Heart rate and oxygen saturation were monitored in the control room remotely using a Nonin 8600 pulse oximeter (Nonin; Plymouth, MN). A video camera was also placed at the opening of the scanner bore for visual monitoring of the rhesus macaque on a screen in the control room. Each rhesus macaque was positioned supine on the scanner bed and the head was centered in the RF coil. A heated saline pack and blankets were used to help maintain the body temperature and animal position during the scan. Oxygen was delivered in proximity to the nose at a rate of 0.5–1.0 L/hr to maintain oxygen saturation >90%. A vitamin E capsule was used as a fiducial mark on the left side of the head during scanning.

MRI data were acquired using a 3T Siemens Trio scanner with a circularly - polarized, 8 - channel dedicated RF head coil with an internal diameter of 18.4 cm (Litzcage, Doty Scientific; Columbia, SC). At each age, a high resolution T1 - weighted magnetization prepared rapid acquisition gradient echo (MP - RAGE) 3D MRI sequence was collected in the sagittal plane (slices = 192; slice thickness = 0.70 mm; number of excitations (NEX) = 1; repetition time (TR) = 2200 ms; echo time (TE) = 4.73 ms; inversion time (TI) = 1100 ms; flip angle = 7°; field of view (FOV) = 180 mm; matrix = 256×256). The total scan time for this sequence was approximately 19 minutes. Additional sequences were also employed but are not reported in this manuscript.

Upon completion of the scans, propofol was discontinued. The total time of sedation ranged from 60 to 90 minutes. During recovery from sedation, the infants were given subcutaneous fluids with 5% dextrose in order to rehydrate and elevate blood glucose levels following fasting. The infants also had access to glucose-enriched water in their incubators. Each macaque was transported back to the CNPRC following the scan and returned to their mothers, and then with their mothers they were returned to their home enclosures at 1, 4, 8, 13, and 26 weeks of age. Each rhesus macaque was returned directly to their home enclosures at 39, 52, 156, and 260 weeks of age.

### Neuroimaging Pipeline

All MRI processing was carried out using an Apple iMac computer running Mac OSX 10.8.2 with 4GB RAM (Apple, Inc.; Cupertino, CA). Annotated pipeline codes used during the course of this experiment and described in this report are freely available and publicly hosted at http://mrhunsaker.github.io/NeuroImaging_Codes/. T1 images were selected for this segmentation pipeline over other collected sequences because the quality of the T1 images was maintained across ages better than other sequences. Additionally, the manual segmentation protocols being used within our laboratory were developed using T1-weighted scans. The protocols below do work for T2-weighted scans, so long as care is taken at each processing step to verify the quality of the result.

### MRI Preprocessing

As a first step, the DICOM images were converted into gzipped NIfTI-1.1 [.nii.gz] format using the dcm2nii tool in MRIcron (http://www.mccauslandcenter.sc.edu/mricro/) using the following terminal bash command:


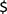
 dcm2nii -a n -g y -f y -n y -e n -i y <DICOM directory>

Of the three outputs from the dcm2nii pipeline, the cropped output (resulting file containing a *-co* prefix) was selected for further processing as dcm2nii automatically cropped out the primate’s neck and shoulders.

Next, the scans were aligned along the anterior and posterior commissures (AC-PC alignment) by using a rigid transformation [brain could only be rotated and translated, but never warped, stretched, or compressed] to a previously manually aligned, age-appropriate template brain using the Advanced Normalization Tools package (ANTS; http://stnava.github.io/ANTs/; [16]). This was accomplished using the following commands with <experimental> referring to the experimental image being aligned to the <template> using a mutual information similarity metric to guide the registration:


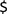
 ./ANTS 3 -m MI[<template>.nii.gz,<experimental>.nii.gz,1,32] -o <output> -i 0–do-rigid true


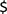
 ./WarpImageMultiTransform 3<experimental>.nii.gz <output>.nii.gz <output>Align.txt -R <template>.nii.gz

The next preprocessing step was to minimize the influence of the bias field signal obscuring grey/white matter boundaries on the registration algorithms. We implemented the N4ITK bias field correction methodology using ANTS [24] with the following three commands; the <input> for each call being the <output> from the previous process as recommended by the developers:


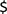
 ./N4BiasFieldCorrection -d 3 -i <input>.nii.gz -o <output>.nii.gz -s 8 -b [200] -c [50×50×50×50,0.000001]


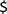
 ./N4BiasFieldCorrection -d 3 -i <input>.nii.gz -o <output>.nii.gz -s 4 -b [200] -c [50×50×50×50,0.000001]


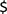
 ./N4BiasFieldCorrection -d 3 -i <input>.nii.gz -o <output>.nii.gz -s 2 -b [200] -c [50×50×50×50,0.000001]

The final preprocessing step was to re-slice the AC-PC aligned images using cubic interpolation and to define a standardized field of view using the convert3d tool bundled with ITK-SNAP (http://www.itksnap.org/pmwiki/pmwiki.php?n=Convert3D.Convert3D) using the following command:


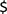
 ./c3d <input> -interpolation Cubic -resample-mm.35×.35×.35 mm -trim-to-size 256×256×256vox -verbose -o <output>

### Semi-Automated Hippocampus Segmentation

To segment the primate hippocampus at all ages, a semi-automated pipeline from ANTS that involves diffeomorphically warping a template brain with fully labeled hippocampus to an individual experimental brain using partial labeling [22] was adopted. The primary strength of this semi-automated pipeline is that it does not require extensive neuroanatomical on the part of the end user. This is important since one of the difficulties in hippocampus segmentation protocols is the time required to adequately train multiple individuals to trace with a consistent level of expertise. However, the placement of limiting markers and the interpretation of any resulting segmentations does require a modest level of training in hippocampal neuroanatomy.

**Figure 1:**
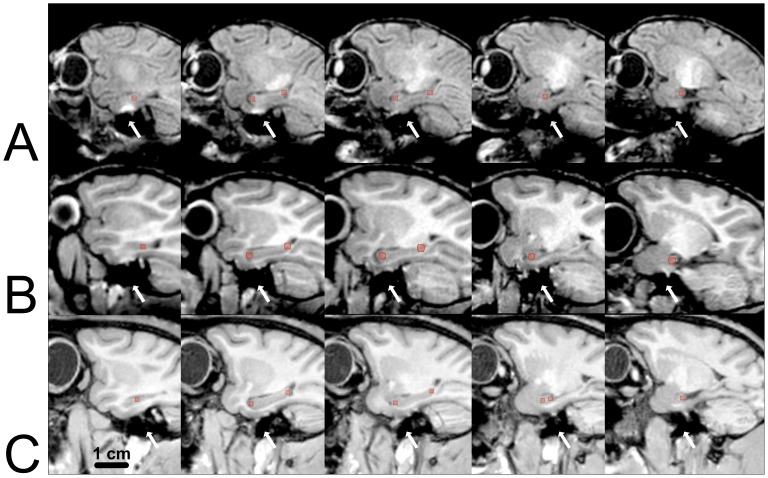
Diagrammatic representation of the partial labeling protocol used in the present study. Sagittal views of landmarks placed every 4th section through the hippocampus are shown for **A.** 1-week-old, **B.** 39-week-old, **C.** 260-week-old rhesus macaque. Note the presence of a susceptibility artifact between brain and the floor of the skull at the level of the entorhinal cortex (white arrows). This artifact was present in all scans. However, it did not interfere with the hippocampus segmentation protocol at any age. Scale Bar = 1 cm.

The partial labeling protocol was modified from the original protocol as follows: Instead of using the six 3D landmark points as in the original study, we modified the protocol to err on the side of systematically, yet sparsely, adding a greater number of landmarks (Figure 1). The first landmark was placed using Multi-image Analysis GUI (Mango; University of Texas Health Science Center; San Antonio, TX) in the sagittal plane at the most lateral section in which the hippocampus was clearly differentiated from the temporal horn of the lateral ventricle. Subsequently, two landmarks were placed on every fourth section of the hippocampus (sagittal separation between subsequent landmarks was 1.4 mm) at the most rostral (anterior) and the most caudal (posterior) extent of the hippocampus. Special care was taken to place a landmark on the medial-most section of the uncus (the most rostral and medial portion of the hippocampus) regardless of spacing from the other landmarks. Between 10–14 landmarks were placed in each hippocampus, depending upon the age of the primate at the time of the scan. Once completed, the landmark points were propagated 1 slice in each direction (*i.e.*, instead of being placed on 1 slice, the landmarks were now 3 slices thick). Dilating the 2D landmarks in this manner was done to make the 2D landmarks placed in Mango more similar to the 3D landmarks placed using the 3D ROI tool in ITK-SNAP by Pluta et al. [22].

Mango was used for the present study rather than ITK-SNAP because our lab had previously compared ANALYZE and Mango for manual tracing regions of interest and found each to return similar segmentation volumes. Additionally, Mango seemed to be the more stable program when handling the MRI scans resulting from the preprocessing pipeline. Preliminary work directly comparing the performance of Mango and ITK-SNAP did not identify any differences between the two programs for placing landmarks, so long as the landmarks were dilated in Mango so as to be similar to those placed in ITK-SNAP.

Once the landmarks were placed in the hippocampus, an age-appropriate atlas brain with a manually segmented hippocampus was diffeomorphically warped with each of the experimental brains. A fully labeled hippocampus was then mapped onto each experimental brain using the landmarks to specifically guide the registration of the hippocampus. The shell script containing the landmark matching protocol and documentation is freely available and publicly hosted at http://github.com/stnava/ANTs/blob/master/Scripts/guidedregistration.sh. Briefly, this script calls a bidirectional, nonlinear diffeomorphic warping of the template (<template>) to the experimental (<experimental>) brain. This warping algorithm specifically uses the landmarks (<experimental_landmarks>) as a guide for warping the template hippocampus (<template_roi>) into the space of the <experimental> image. The template hippocampus is then warped into the space of the experimental image.


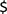
 sh ./guidedregistration.sh <template>.nii.gz <template_roi>.nii.gz <experimental>.nii.gz <experimental_landmarks>.nii.gz <output>_hippocampus 100×100×10 3

For later processing, the hippocampus segmentations need to be in 8 bit rather than 32 bit floating point format. So, we used the following command in convert3D to make the conversion and binarize the segmentation (hippocampus mask = 1, background = 0) with the input into this script being the segmented hippocampus that was the output from the above process (*i.e.*, <output>_hippocampus from above = <input> in the call below):


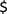
 ./c3d <input>.nii.gz -binarize -o <output>.nii.gz

### Machine Learning Algorithm Based Segmentation Correction

We were able to improve the semiautomatic hippocampal segmentations by using a machine-learning algorithm that corrects systematic errors in semiautomatic segmentations (Automatic Segmentation Tool Adapter; SegAdapter; freely available and publicly hosted at http://www.nitrc.org/projects/segadapter/; [23]). This tool takes advantage of the nature of computers to commit primarily systematic errors, rather than random errors. What this means is that if a series of subjects are scanned using the same sequences on the same MRI scanner, then the errors of any automated segmentation protocol will be similar across brains (*e.g.,* consistent partial volume effects or inclusion of choroid plexus or CSF as hippocampus tissue, etc).

This SegAdapter is trained by providing a number of manually traced, fully labeled hippocampi with a set of semi-automated hippocampal segmentations using the following codes [with the.txt files containing lists of images and corresponding ROIs]:


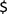
 ./bl ./inputIMAGEFILE.txt ./manualSegmentationFile.txt./autoSegmentationFile.txt 1 2 4×4×4 .1 500 ./TRAINING/SegAdapter

This SegAdapter learning algorithm corrects the semi-automated output from the partial labeling protocol to correspond to the fully labeled manual tracings. In the present experiment, 2 MRI scans (1 male and 1 female) from each age (1,4,8,13,26,39,52,156,260 weeks of age) were used to train the learning wrapper (*i.e.,* a total 18 scans were used for the training of the 216 studies that were available). We specifically *included* cases where there was *poor* signal to noise ratio at all time points or inconsistent signal inversions present in the scans from 1 week-old primates. These imperfect scans were selected so that the machine learning algorithm would have a complex dataset encompassing many different types of segmentation errors from which to develop a template used to correct automatic segmentations. We selected only 2 scans at each age to demonstrate how robust the SegAdapter actually was since a greater number of training images results in better algorithm performance. Preliminary experimentation showed that this algorithm did a more reliable job in adjusting the segmented hippocampi when given a diverse data set than if provided only cases devoid of scanning artifacts. Once the SegAdapter had been trained, each partially labeled hippocampus was corrected by using the following command:


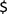
 ./sa <input>.nii.gz <segmented_ROI>.nii.gz./TRAINING/SegAdapter <output>.nii.gz

Once all of the segmentations were obtained, they were compared with manually segmented hippocampi. To evaluate the goodness of fit of the hippocampus segmentation that was acquired through the partial labeling protocol as well as the segmentation corrected by the SegAdapter learning algorithm, spatial overlap (DICE) was computed using convert3d with the following commands, with “*1*” referring to the label of the segmented hippocampus:


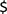
 ./c3d -verbose -overlap 1<input>.nii.gz <segmented_ROI>.nii.gz <Manual_ROI>.nii.gz >> <output>.txt

Specifically, the DICE overlap is calculated as 2× the overlapping voxels divided by the total number of voxels from the semiautomated segmentaion (A) and the result of the SegAdapter correction (B) (*i.e.*, 2(A∩B)/(A+B)).

### Manual Hippocampus Tracing

All of the hippocampi evaluated in the present study were manually segmented by a single trained experimenter as part of a separate study (MRH; Hunsaker et al., *in revision;* the hippocampus manual tracing protocol is publicly hosted at http://mrhunsaker.github.io/Hippocampus_Protocol). For each scan, the hippocampus was manually traced using Mango. To control for any effects of hemispheric bias (*i.e*., right volumes being larger than left volumes), the second time the images were traced they were converted from neurologic to radiologic space. The intra-rater reliability across these tracings was maintained at >.86. The hippocampal volumes for the right and left hippocampi traced in Mango were exported into a.csv file for later analysis and the ROIs saved in gzipped NIfTI-1.1 format. Each of the volumes of the individual regions of interest were quantified in the FMRIB Software Library (FSL v5.0; http://fsl.fmrib.ox.ac.uk/fsl/fslwiki/; [25]) with the following command:


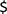
 ./fslstats <input>.nii.gz -V >> <output>.txt

For three-dimensional visualization of the reconstructed hippocampus, the regions of interest were imported into MRIcroGL (http://www.mccauslandcenter.sc.edu/mricrogl/) and Mango for 3D rendering.

## Results

### Partial Labeling Protocol

**Figure 2:**
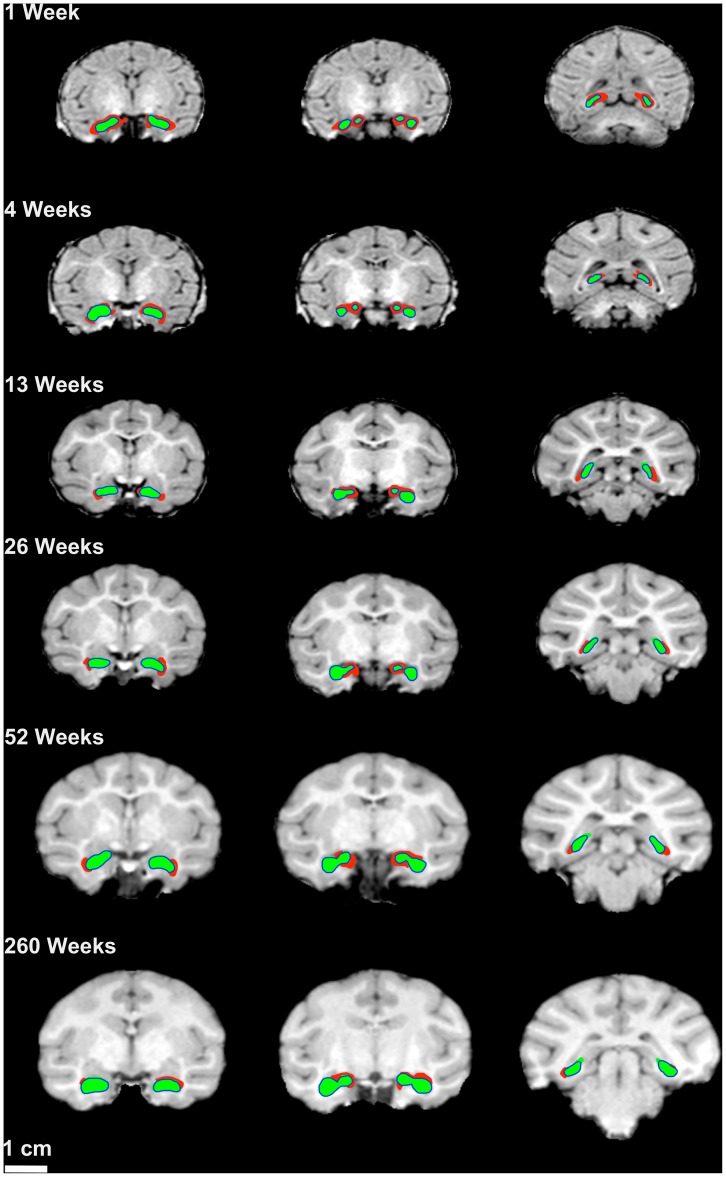
Example segmentation for of the partial labeling protocol across ages. The coronal slices are from the same primate at 1 week, 4 weeks., 13 weeks, 26 weeks, 52 weeks, and 260 weeks of age. In red is the landmark guided partial label segmentation. In green are results of the SegAdapter adjustment. The outlines in blue are the manual segmentations for these sections. Note that the segmentation error (in red) appears to be systematic across ages. This error is primarily due to erroneously segmenting temporal horn as hippocampus. Scale bar = 1 cm.

The partial labeling protocol was able to segment the hippocampus within a very reasonable tolerance (Table 1 and Figure 2). However, there was a systematic inclusion of white matter ventral to the body of the hippocampus as well as a small amount of inclusion of the temporal horn of the lateral ventricle (Figure 2). In one 26 week-old scan, a small area of temporal cortex located lateral to the body of the hippocampus was misclassified as hippocampus.

**Table 1:** Comparison of Hippocampus Segmentation Methods.

Age	Manual v Semi-Automated Label	Manual v SegAdapter	Manual Reliability
1 week	.68+/−.13	.86+/−.08	.88+/−.10
4 week	.73+/−.09	.89+/−.09	.92+/−.12
8 week	.74+/−.14	.91+/−.06	.92+/−.07
13 week	.69+/−.10	.93+/−.09	.94+/−.08
26 week	.77+/−.08	.90+/−.07	.93+/−.11
39 week	.74+/−.06	.92+/−.05	.96+/−.14
52 week	.78+/−.09	.94+/−.09	.95+/−.09
156 week	.70+/−.14	.91+/−.10	.94+/−.10
260 week	.79+/−.13	.95+/−.09	.97+/−.11

Average DICE coefficients for semi-automated segmentation and SegAdapter methods compared to manually segmented hippocampi. Data are from 24 rhesus macaques, 12 male and 12 female. Error given +/− standard deviations from the mean (sd).

For the present experiment, placing the landmarks in the hippocampus of each of the rhesus macaque scans took between 5 and 10 minutes. Overall, the MRI preprocessing steps took 3.25 hours for each brain. The guided registration script in ANTS took approximately 38 minutes of computational time for each brain.

### SegAdapter Algorithm

**Figure 3:**
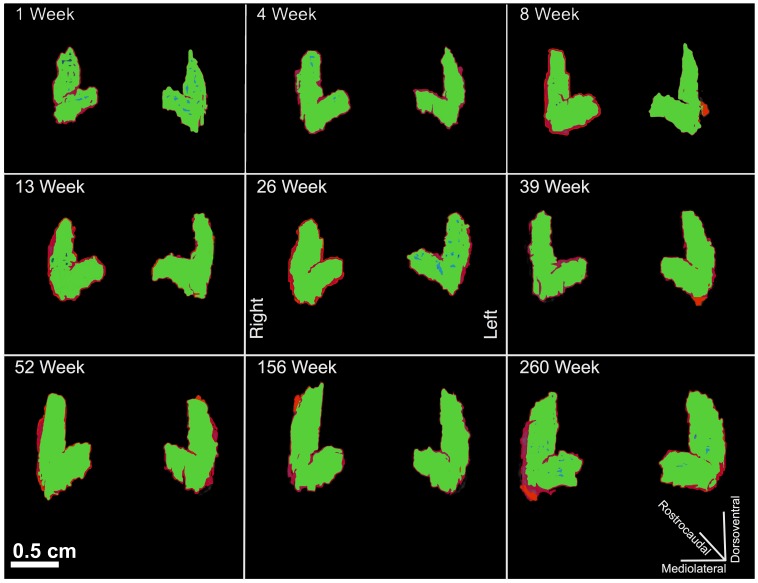
Example Renderings of hippocampi segmented via the partial labeling protocol and SegAdapter corrections 1 week, 4 week, 8 week, 13 week, 26 week, 39 week, 52 week, 156 week, and 260-week-old rhesus macaque. Red renderings are the semi-automated hippocampus segmentations and green renderings are SegAdapter corrected hippocampus segmentations. Manual tracings are shown in blue, and are obscured due to high overlap with SegAdapter segmentations. Scale Bar = 0.5 cm.

**Figure 4:**
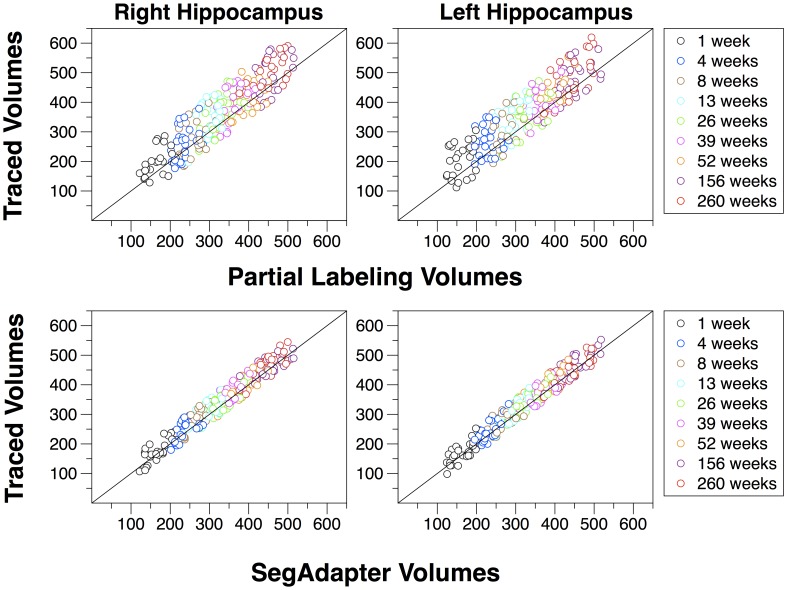
Plots of Hand Traced manual hippocampus segmentations compared to the results of Partial Label guided semiautomated segmentation as well as SegAdapter corrected volumes. Note the Partial Labeling consistently over-estimated hippocampus volumes, whereas the SegAdapter results fall along the unity line with manually segmented volumes.

The SegAdapter was able to correct the vast majority of the systematic errors that resulted from the partial labeling protocol and semi-automated segmentation pipeline (Figure 2). The subhippocampal white matter, temporal horn, and extra-hippocampal voxels were now excluded from the tracing and the overall region traced was smoother and appeared more visually similar to a manually traced hippocampus (Figure 2, 3). The similarity between SegAdapter corrected and manual segmentations is confirmed by the high DICE (spatial overlap) coefficient that approached, but remained lower than the intra-rater reliability results (Table 1). Example of manually segmented hippocampi compared to the results of the Partial Label guided segmentation as well as the SegAdapter corrections are shown in Figure 4.

For the present experiment, final training of the SegAdapter took approximately 22.5 hours of computational time. Once the SegAdapter had been trained, it took under 3.5 seconds to correct each partially labeled hippocampus. The training set used for training the algorithm was available from an earlier experiment, but took approximately 25.5 hours to hand trace the pairs of hippocampi from the 18 rhesus macaques used in the training set.

There was a dramatic overall temporal benefit of this method over a complete hand tracing study. Tracing all the hippocampi included in this report took approximately 540 hours for the first pass, and they were traced a second time for reliability analyses, resulting in approximately 1080 person hours to reliably acquire the hippocampal ROIs. This did not include the initial reliability training, which required another 20–25 hours. The preprocessing steps reported above were identical for the two experiments. The present method required substantially less time, and the computational time was not limited to the workday, as scripts could be run overnight or whenever the computer was not in use.

## Discussion

The goal of the current study was to develop an easy to use processing pipeline that would be accessible to any research laboratory to partially automate the segmentation of anatomical regions of interest. The first goal was to use freely available tools that had similar dependencies and did not rely upon any commercial software packages to implement. The second goal was to develop a pipeline that would facilitate consistent data across laboratories by removing experimenter bias as much as possible without sacrificing anatomic rigor. Although not explicitly stated at the outset, a related goal was to minimize experimental time as much as possible.

This effort was implemented by applying tools that depended primarily on the platform independent ITK image-processing library and more particularly on ANTS, a neuroimaging toolkit developed as a simplified program that lets the end user take advantage of ITK without having to acquire specialized knowledge or learn extensive computer coding skills. All of the software packages used in this protocol are freely available, open source packages that remain under active development, and primarily depend upon the Insight Segmentation and Registration Toolkit (ITK), a freely available cross-platform image analysis library. By using these tools, we were able to segment the rhesus macaque hippocampus from MRI scans collected as early as 1 week of age and out to 260 weeks of age.

The strategy for hippocampal tracing was applied to a large database of longitudinal MRI scans of 24 rhesus macaque monkeys (12 male and 12 female). Nine scans were available from each monkey from 1 to 260 weeks of age, for a total of 216 MRI scans to segment. Each of the MRI scans had previously been manually traced using a rigorous protocol by a single reliable tracer as part of a separate study (Hunsaker et al., *in revision*; http://mrhunsaker.github.io/Hippocampus_Protocol/).

We have presented a validation of a robust method for highly accurate semi-automated segmentation of rhesus macaque hippocampus from 1 week to 260 weeks of age. By applying a machine correction algorithm to eliminate systematic errors in the semi-automated segmentation, it is clear that these methods are capable of producing hippocampal masks that approach the same levels of anatomic rigor and level of quality as segmentations generated by gold-standard manual tracers. Even MRI scans from rhesus macaques as young as 1 week of age were reliably segmented using these protocols. This is not a trivial outcome since there was often either very little gray/white matter contrast or a complete gray/white matter signal inversion that made hand tracing difficult. Despite this signal inversion, the fact that the semi-automated segmentation protocol and landmark guidance relied on manual placement, these methods appeared to be sufficient to reliably place the hippocampus on each individual scan and the SegAdapter algorithm was able to correct most of the systematic errors introduced by the small differences in the MRI scans, as demonstrated in Figure 2.

During the development of this pipeline, we found that a more complex training set (*i.e.*, a training set with suboptimal signal to noise, signal inversion, artifacts, etc.) results in better corrections than a less complex set. This is presumed to occur because the SegAdapter algorithm is capable of learning the full complement of systematic errors that result from the diffeomorphic hippocampus-warping algorithm. By training the algorithm on as diverse a training set as possible, more of the systematic errors were identified and corrected. If a pristine, error free dataset were used for training, then the learning algorithm would not have sufficient errors to identify, and thus would fail to correct errors resulting from the semi-automated segmentations. We observed this phenomenon in our preliminary experimentations, similar to what Wang et al. [23] have reported. Although a larger training set provides more reliable segmentations, we chose 1 male and 1 female from each age as a training set to maximize the quality of the segmentations while minimizing the computational time spent to train the machine learning algorithm.

It is further notable that the methods employed within this manuscript were originally developed and validated in preclinical human neuroimaging research (*cf.,* [15], [16], [17], [22], [23]). This study has validated these protocols for the nonhuman primate – even for scans that were carried out in very young rhesus macaques. The relative ease by which these tools were able to work for the primate scans suggests that they could facilitate cross-species comparisons not only using the same suite of tools, but also exactly the same functions within those tools (*cf.,* [26]). This will undoubtedly improve cross-species comparisons of developmental or degenerative trajectories.

A further benefit of the present pipeline, as opposed to using other freely available tools such as Freesurfer, is the inherent flexibility in the methodology. Freesurfer does not always reliably perform segmentations, particularly in the presence of abnormal or pathological anatomy (*cf.,* [15], [16], [17], [23]). Moreover, to date there is no reliable way to modify Freesurfer for the segmentation of rhesus macaque or rodent MRI data, which are relatively straightforward using the present pipeline.

An additional benefit of this approach is the possibility for extension into different regions of interest within the brain. If a researcher has a template for any region of interest (or multiple regions), the only requirement for these methods to work is the careful determination of where to place landmarks. The present protocol is currently being applied to provide concurrent hippocampus and amygdala segmentation, with relatively high reliability with manual tracings of both neuroanatomical loci, at least in primates older than 39 weeks of age. By extension, if researchers have maps for cortical regions of interest (*e.g.*, the UNC Primate Brain Atlas available at http://www.nitrc.org/projects/primate_atlas/; [27]), so long as systematic landmarks can be placed across brains, it is possible to increase the reliability of diffeomorphic warping of the cortical tracings onto experimental MRI scans.

While neuroanatomical expertise is essential for interpreting changes in neuroal structures, the present method requires rather modest neuroanatomical training. In the present report, only a single trained hippocampal tracer was required. Manually segmented hippocampi were required to provide a training set for the SegAdapter pipeline. Once this training set is established, all that is required is for another experimenter to place landmarks on the hippocampus in a very clear, repeatable manner that does not require extensive neuroanatomical expertise. Specifically, as long as landmarks are placed consistently in the hippocampus, the partial labeling protocol is able to segment the hippocampus. The consistent difference is that not placing a landmark on the most lateral aspect of the hippocampus results in a small amount of hippocampus tissue segmented as temporal horn rather then as hippocampus. Importantly, so long as the actual MRI scanner hardware and the scan sequence are maintained, additional contributions from the neuroanatomist other than basic quality control during pilot experiments are unnecessary.

One limitation of the tools used in the present study is the amount of computational time required to perform the necessary transforms. Using a computer system with 4 GB of RAM, it took on average 2.25 hours/brain to perform the N4ITK bias field correction, approximately 4.75 hours per brain to perform the semi-automated hippocampus segmentation, and approximately 22.5 hours to train the SegAdapter using 18 scans (longer if the pilot experiments are taken into account). All other steps took under 2 minutes per brain. This limitation can easily be overcome with access to a computer with a greater amount of RAM, cloud computing, or distributed cluster computing systems since the pipeline made heavy use of ANTS and ITK, which are optimized for distributed computing (*cf.* [16], [23]). Despite this limitation, the method reliably performed as well as manual segmentations. These methods also required far fewer trained personnel, and took substantially less time to acquire hippocampus ROIs than manual tracing methods.

In summary, we describe a simple protocol and provide sample code for semi-automatic hippocampus segmentation in rhesus macaque monkeys from the early postnatal period into adulthood (1 week-260 weeks). This method requires very little neuroanatomical expertise and can be performed using commercially available, off the shelf, computational resources and public domain software. This protocol results in hippocampus segmentations that show reliably high DICE overlap with manually segmented hippocampi. Virtually any laboratory can easily apply this method with access to MR Images; the only limiting factor is the computer processing time. Further developments in the tools used in this pipeline may serve to mitigate this limitation and increase the utility of this semi-automated pipeline, opening a door to increased throughput of MRI based anatomical research.
